# *“A disembodied voice over the telephone*”: a qualitative study of healthcare practitioners’ experiences in geriatric medicine

**DOI:** 10.1186/s12877-023-03909-y

**Published:** 2023-05-05

**Authors:** Frankie Brown, Isabella Sanders, Ross Watkins, Elisabeth Grey, Paula Smith, Daniella Springett, Tomas Welsh, Fiona Gillison

**Affiliations:** 1 Department for Health, University of Bath, Bath, BA2 7AY UK; 2 Department of Psychology, University of Bath, Bath, BA2 7AY UK; 3 Research Institute for the Care of Older People, RICE, Royal United Hospitals Bath NHS Foundation Trust, Bath, BA1 3NG UK

**Keywords:** Telehealth, Remote consultation, Communication skills, Access to care, Patient preference

## Abstract

**Objectives:**

This study explored the experience of delivering care remotely among practitioners in a UK geriatric medicine clinic.

**Methods:**

Nine semi-structured interviews were conducted with consultants (*n* = 5), nurses (*n *= 2), a speech and language and an occupational therapist, and thematically analysed.

**Results:**

Four themes developed; Challenges of remote consultations; Perceived advantages of remote consultations; Disruption of involvement of family members; Impact on care staff. Participants felt that rapport and trust had been more feasible to develop remotely than they had anticipated, although this was more challenging for new patients and those with cognitive or sensory impairments. While practitioners identified advantages of remote consultations, including involving relatives, saving time, and reducing anxiety, they also experienced disadvantages such as consultations feeling like a ‘production line’, missing visual cues and reduced privacy. Some participants felt their professional identity was threatened by the lack of face-to-face contact, linked to feeling that remote consultations are not suitable for frail older adults or those with cognitive deficits.

**Discussion:**

Staff perceived barriers to remote consultations that went beyond practical concerns, and suggest support for building rapport, involving families, and protecting clinician identity and job satisfaction may be warranted.

**Supplementary Information:**

The online version contains supplementary material available at 10.1186/s12877-023-03909-y.

## Introduction

There has been a need to provide remote consultations since the onset of the COVID-19 pandemic, at a speed and coverage not previously anticipated. While the speed of action needed was problematic in some ways, as the skills and equipment to provide high-quality consultations were not in place at the start of the process, it has provided many clinics with experience of how things may work for the future development of the service. In the UK, a greater use of technology in health services was already planned ahead of the pandemic [1]. The NHS long term plan explicitly states that in ten years’ time ‘*The NHS will offer a ‘digital first’ option for most, allowing for longer and richer face-to-face consultations with clinicians where patients want or need it.*’ (NHS, 2019). The aim of this study is to explore what can be learned from the experience of running a remote clinic to deliver geriatric medicine during the first year of the COVID-19 pandemic.

Clinicians’ perceptions of remote consultations are a critical factor in determining their acceptance as a model of care [2]. When remote models of care are appropriately designed and delivered, support can be high: For example, a study conducted in a large general outpatients department (of which 20% of patients were > 65 years old) reported that the vast majority of clinicians (84%) agreed or strongly agreed that they could provide the same quality of care as a face-to-face visit, therefore, nearly all (97%) clinicians agreed they would use remote consultations again [3]. High levels of clinician satisfaction with remote consultations have also been reported in a range of specialised hospital departments during the COVID-19 pandemic (> 75% satisfaction) [4]. There appears to be increased satisfaction with the additional visual component provided by a video consultation above telephone consultations [5–7], primarily due to the increased ability to build rapport with patients and pick up on the visual cues via video. The most frequent barriers to the adoption of remote consultations worldwide prior to the COVID-19 pandemic were technology-related challenges [8], which have continued to present a substantive barrier according to more recent research [9]. However, little is known about the impact of other factors on acceptability, including the changing patient-professional relationship in remote consultations, and how this will impact the health care professional’s perception and acceptance of the continued use of remote consultations as a model of care.

Geriatric medicine is an area where there are particular challenges for remote consultations due to the high prevalence of sensory and cognitive impairments, and functional difficulties among patients [10–12]. For example, during the COVID-19 pandemic in a geriatric outpatient clinic in an Australian hospital, half of the remote consultations needed to be rescheduled due to language barriers, poor telephone/internet connections, patients having hearing difficulties, inability to perform assessments (e.g. visualise a tremor, assess gait, perform a cognitive assessment) or treat the patient (e.g., wound management) [13]. As a result the mean overall geriatrician satisfaction with remote consultations (telephone and video) was reported as 5.9 out of 10 [13], which is significantly lower than the 88% of physicians satisfied with remote consultations (video) reported pre-COVID 19 pandemic [14]. Better understanding of what underpins poorer job satisfaction with remote consultations will be important in designing future remote services specifically for these conditions and patients.

The aim of this study was to explore the experience of health practitioners in delivering remote care during the first year of the COVID-19 pandemic in a UK hospital. In particular, we aimed to explore the characteristics of, and barriers and facilitators to more successful and satisfactory consultations.

## Methods

### Ethics

The study was approved by the Research Ethics Approval Committee for Health at the University of Bath (Reference Number: EP 20/21 008). An invitation was sent from the management team to all the healthcare practitioners working within the department of Geriatric Medicine with information about the study, advising them to contact the research team if they wished to participate. Informed consent was sought and documented electronically prior to each interview. Participant interviews were audio recorded, transcribed verbatim, and anonymised by assigning each participant a unique reference number (e.g., GC01) and a pseudonym for attributable quotes.

### Participants

Healthcare practitioners working within the department of Geriatric Medicine in a large District General Hospital in the UK were invited to take part. In order to capture maximum variation in the experiences of healthcare practitioners in the department a maximum variation sample was recruited using a combination of purposive, convenience and snowballing techniques [15]. Recruitment continued until we had recruited staff with a mix of role type (including consultants, nurses and healthcare therapists), seniority, age and gender.

### Procedure

Volunteers provided data on their demographics including age group, gender, ethnic group, role, and length of time in the profession prior to interview. One researcher (RW), experienced in qualitative health services research, undertook all interviews using Microsoft Teams between February and March 2021, which lasted between 45 min and one hour. The interviews focussed on participants’ experiences of conducting a consultation remotely (via telephone or online) according to a topic guide (Additional file 1: Appendix one). The questions were designed to be open-ended and broad, while focussed on the topic in order to elicit rich responses [16]. The topic guide was modified throughout data collection to incorporate relevant topics identified in earlier interviews. This is consistent with an inductive approach in which theory is derived iteratively and develops through the analysis of data. Memos were taken during the interview process to enable the authors to determine characteristics of participants, which provided further insight into potential themes [16].

### Data analysis

Participant interviews were audio recorded and transcribed verbatim. Transcripts were analysed thematically [17, 18] using NVivo software (QSR International; Version 12 Pro) to help organise, code and explore the data. The focus of the analysis was to organise the data in a meaningful way according to the a priori aims of the study, as well as to allow identification of topics and issues of importance to participants. Coding was conducted by IS, following which IS and FG used a framework approach [19] to support the systematic analysis of data around the research questions. Further interpretation and discussion culminated in the creation of a thematic resource document. This reported all the relevant coded data under overarching themes or headings. The themes, their names and explanations were continually refined through discussion between the researchers to ensure that they were distinct from other themes, internally coherent and consistently applied.

## Results

Nine interviews were conducted with regular clinical staff at a single geriatric medicine clinic, five with consultants, two with nurses, one with an occupational therapist and one with a speech and language therapist (Table 1). The majority (89%) of participants were white, 63% were female, and ages ranged from the age bracket 25–34 to the age bracket 45–54 years. Participants had worked their respective professions from 5 to 20 years. To preserve anonymity, quotes are attributed to either consultants or care team members. Participants were asked to describe their use of video and telephone appointments following the first lockdown, with the majority of participants engaging with patients via the telephone or both. Only one participant conducted consultations solely through video.

**Table 1:** Participant demographics

Profession	Gender	Age (years)	Time in profession (years)	Ethnic Group
Consultant	Male	45–54	5	Mixed ethic background
Consultant	Male	45–54	20	White
Consultant	Female	35–44	8	White
Consultant	Male	35–44	15	White
Consultant	Male	45–54	16	White
Nurse	Female	45–54	9	White
Nurse	Female	45–54	14	White
Speech and Language Therapist	Female	25–34	5.5	Mixed ethnic background
Occupational Therapist	Female	35–44	10	White

Four themes were developed with a view to highlighting issues specific to those working with older patients or patients with complex cognitive impairments: Challenges of remote consultations; Perceived advantages of remote consultations; Disruption of involvement of family members; Impact on care staff (Table 2).

**Table 2:** Themes and sub themes

Theme	Sub-themes
1. Challenges of remote consultations	1.1 Difficulties in establishing rapport with patients
	1.2 Loss of information from informal interactions
	1.3 Importance of communicating bad news in person
2. Perceived advantages of remote consultations	2.1 Reducing structural constraints
	2.2 Benefits to patients
3. Disruption of involvement of family members	
4. Impact on care staff	4.1 The patient-professional relationship
	4.2 Professional identity
	4.3 Confidence in doing the job well

## Theme 1: Challenges of remote consultations

### Difficulties in establishing rapport with patients

Participants considered that building and maintaining rapport was more challenging in remote than face-to-face consultations, but most agreed it was still possible. Initial consultations when calling an unknown patient were considered the most difficult, although participants talked of how they had successfully found ways to adapt their approach.

*…new consultations are a bit more challenging, but I think you still establish rapports in different ways. You use the same terms of phrase, you still gain the patient’s confidence, you still use enabling open questions and neurolinguistic stuff to try to get the best things out of them on the telephone.*

*(Consultant Geriatrician)*

Many shared the sentiment that remote consultations felt impersonal which was particularly undesirable for participants who would be involved in a patient’s care longer term.

*And because I’ve been able to see people face-to-face and that’s always going to be my first choice, because then I build up a relationship with somebody. And if I’m going to be looking after their Parkinson’s for the next 10 years, 12 years, I want a relationship with them, which I feel probably isn’t going to be quite the same if I do it like this [remotely] than if I actually see them face-to-face.*

*(Consultant Geriatrician)*

Similarly, participants felt it was more difficult to build trust with the patient remotely.

*It’s very important to be face-to-face, not just for identifying what might be wrong with them, but getting their trust as well, and them getting trust in you and you understanding them.*

*(Consultant Geriatrician)*

### Loss of information from informal interactions

Many participants referred to the lack of more intangible information in remote consultations, that can often be important for clinical assessments. For example, assessing disease progression which may be informed by visual cues outside of a formal clinical examination, such as the example below relating to Parkinson’s disease:

*Yeah, I mean we don’t do, I mean it’s just really seeing somebody walk into a room, or how they interact with you when you go up and speak to them. Do they automatically shake your hand, this is all part and parcel of your assessment, which is lost when you’re doing something remotely. They seem silly little things which you wouldn’t normally notice, but even the way they hold the pen, what their body’s like.*

*(Care Team Member)*

The importance of conversations which were not necessarily part of the assessment were also highlighted as giving insight into aspects of the patient life that may not be obvious from asking traditional questions. Participants felt that these conversations usually occurred incidentally, when for example nurses were carrying out blood pressure tests.

Others reported how it was hard to provide comfort to patients remotely, as one of the care team members states ‘*there’s nothing like the use of touch with somebody’*. Even among participants who were confident that empathy could be conveyed over video through vocal and facial expressions if needed, there was a shared sentiment that the *‘ultimate experience for the patient is probably having the clinician in the same room as you’ (Consultant Geriatrician).*

### Importance of communicating bad news in person

Not all participants considered remote consultations to be inferior to face-to-face, as long as offered to appropriate patients at appropriate stages of treatment. However, in addition to initial consultations, there was consensus that the communication of bad news, an initial diagnosis or worsening condition, should be done face-to-face. Participants described difficulties in conveying empathy adequately when not present in person, recognising the emotional weight that a poor diagnosis places on patients and relatives.

*Giving someone a diagnosis of a neurodegenerative condition that is a terminal diagnosis, it shortens your life expectancy and means you’re going to be on medication forever [and] that probably has, probably will touch almost all aspects of your life, including your cognition, I feel is not something I can do with people when I’ve only ever spoken to them on the telephone. I feel that if I’ve seen them and getting that sort of news, bad news over the phone is 10 times worse.*

*(Consultant Geriatrician)*

## Theme 2: Perceived advantages of remote consultations

### Reducing structural constraints

Interviewees identified some potential advantages of remote consultations, including convenience and reducing organisational pressures such as space:

*There’s an ease, because [if] I have to cancel an afternoon clinic because I can’t do that for whatever reason and I [can now] go “well it’s all right, I can’t do it in the afternoon, but I can do it in the morning”. Now before that [it] would be “I can’t do that” because I can’t have the clinic room and the nurses and all the rest of it. But actually it doesn’t matter, I have the clinic wherever I like. I can be anywhere now, all I need is a computer and a phone and I’m done.*

*(Consultant Geriatrician)*

Another advantage identified was when it is useful to share visual images of scans and charts for discussion.

*Actually one thing that’s been really good is being able to screen share … Quite a big part of our therapy is looking at these things, because we see quite a lot of people with psychogenic swallowing issues, where part of the rehab is around really showing them what is or isn’t going wrong with their swallow. … it works really well, because to do it remotely, because they’ve got it in front of them. You’re not huddled round this laptop and relying on your rubbish signal out in the sticks or whatever.*

*(Care Team Member)*

### Benefits to patients

There was also general agreement that patients felt more relaxed in their own homes surrounded by familiar items and relatives or carers. This could assist the flow of conversation between participants and patients, reducing some of the anxiety patients may feel being in a clinic.

*Because I think with the best will in the world often people do feel a little bit anxious coming to a clinical setup and thinking I have to talk about my condition and this person’s making notes. Whereas if they’re just sat at their kitchen table with a cup of tea I think hopefully they do feel a little bit more relaxed and maybe more able to share things that they may not have shared [at the clinic].*

*(Care Team Member)*

However, some participants reported a feeling that the more relaxed setting sometimes led patients not to value remote consultations as much they did face-to-face appointments. Some care staff perceived this to result in a higher rate of non-attendance or poorer engagement in the process.

*…maybe that goes back to the informality, people think oh well it’s only a little chat on a video, that’s not a big deal, it’s not as big a deal as a proper outpatient appointment. So yeah, I do think people may be like oh well never mind, I couldn’t do it so I’ll forget it.*

*(Care Team Member)*

## Theme 3: Disruption of involvement of family members

The impact of remote consultations on the participation of family members in appointments was mixed. Having family members present during consultations was identified by participants as important to patients for emotional support, assisting with communication if patients were hard of hearing and for participants to consult with family members about future treatment or care plans. As such, if remote consultations meant that relatives were less likely to attend (e.g., it is harder to involve others in a phone call than a video call) clinic staff felt this was a negative consequence. However, using video or telephone appointments could be a positive step in enabling family members to be part of their relatives’ care despite living far away, and in such cases provided a new *‘gold standard’*, compared with face-to-face appointments.

*But as I said before if it’s a relative a long way away, I think it’s quite valuable because they’ve necessarily been pulled in; whereas historically in that sort of scenario we’d probably have seen the patients, and we might have phoned or spoken to the relative at another time, but we wouldn’t have had the immediate three-way discussion.*

*(Consultant Geriatrician)*

Participants commented that in face-to-face clinics when relatives did attend, health practitioners may have spoken to them separately bringing further understanding of the true picture at home, an element that is difficult to replicate virtually.

*There’s the limitation that you’ve always got that patient and husband or wife or whoever you don’t ever get any separate time with either of them, it’s never very private. So maybe we would, we always like to take people off separately actually, because there’s a different story sometimes to that person and how they’re getting on to what they say, and it’s difficult to say that in front of the person with memory problems.*

*(Care Team Member)*

Speaking to relatives separately was something that some clinics and staff had tried to maintain initially when moving to a remote format. However, as it happened less automatically as part of a visit, this had slipped from being part of the routine care for some.

*Initially what we offered was “did you want to have a phone call separately?” I’m just thinking about it now that actually we’ve stopped doing that, I don’t know where that happened but that was the thinking, that’s how we did things initially. But we’re actually not offering that, but actually I should be offering ...that chat separately. And sometimes in the beginning that was taken up actually.*

*(Care Team Member)*

## Theme 4: Impact on care staff

### The patient-professional relationship

A number of participants expressed concern that in moving towards more remote care, there had been a discernible shift in the patient-professional relationship, which in turn impacts professional identity and job satisfaction. This was attributed to being less able to spend time with patients, the lesser opportunity for informal contact with families and wider support services (e.g., having Alzheimer’s Society personnel co-located in the clinic), and beliefs that remote consultations undermine the quality of the communication or contact between people. Some participants admitted that speaking to new patients over the telephone made it difficult to remember them because of the lack of physical connection. Others spoke of the ‘*disembodied voice over the telephone’*, indicating the connection was less human or engaging and some reported that seeing patients remotely could feel like a ‘*conveyer belt’.* Several interviewees reported feeling guilty about these factors and that they were less able to meet their standards of being a ‘good clinician’.

*I think that [remote consultations] can affect your feeling of wellbeing or your confidence in your role….. Does it affect them [patients], their care? By the metrics that people higher up would measure it by, I don’t know, but it definitely changes what it feels like to be in the job for sure.*

*(Care Team Member)*

*There were times I felt that as much as I think I’m quite good at it, didn’t always hit the spot every time if I’m being honest. I mean I got there in the end, because I saw her and then I found out what the problem was.*

*(Consultant Geriatrician)*

There was recognition that some ways in which things had changed were as a result of service reorganisation which may have already been planned prior to the COVID-19 pandemic, but which had sped up as a result. The speed of change rather than the change itself may have made these shifts more visible and less acceptable to staff. This is exemplified by one member of the team who changed their role as a result of disagreeing with the changes to practice initiated by the shift to remote care. While these considerations may be shared across the range of medical specialties, some interviewees felt that this impact was particularly impactful for participants caring for older people:

*I mean my overall feeling is that it’s [remote consultations] more of a problem than a problem-solving tool, particularly for our group of patients. I can see in other people it might be quite valuable, but for our old and frailer patients I’m not sure that it’s the optimum way of doing things really.*

*(Consultant Geriatrician)*

### Professional identity

Overall, the shift in role that participants had noticed as a result of services moving to a remote setting was felt by some to threaten their professional identity by changing the nature of the patient-professional relationship, and taking away some of the elements of the job from which they previously derived job satisfaction. However, not all interviewees felt this way. One consultant who had discussed feeling confident in delivering remote diagnosis and care had very positive views of his video consultations, and did not feel his sense of professional identity or ability to do his job was affected.

*I guess the ideal for me as the lazy clinician is to be sat in a room and everything happens for me. So it’s like so and so is here on the screen, somebody’s doing it, I’m just talking to them, making some notes and everything, the consultation of the future is that voice technology then is writing the notes and putting them into the computer record and I can just give them the advice and then move on. But yeah, for me it’s relatively straightforward. I have few wants and needs and as long as I can see the patient and communicate to them, it doesn’t really bother me.*

*(Consultant Geriatrician)*

### Confidence in doing the job well

Having less opportunity to observe patients led some participants to admit they were more likely to carry out further testing and an increased likelihood of a subsequent face-to-face appointment; this was to allay their concerns that visual cues had been missed in the remote consultation.

*Yes, because what you’re taking, without the seeing the patient, without being able to do, the examination helps drive what is actually maybe the diagnosis, therefore what tests you need; whereas if you’ve got a slightly difficult historian where you can’t quite get what they’re getting at and they’re anxious over the phone, they can’t hear you. You say OK right, I think we should do a brain scan. If I’d seen her first off and examined her, I probably wouldn’t have done a brain scan.*

*(Consultant Geriatrician)*

Many staff noted that running appointments remotely saved clinician, care team and patient time. While this may have enabled participants of different job roles to see more patients per session, it was also experienced as a detriment by others as it reduced the time they had to reflect on individual patients and their care.

*And then not having that travel time as well to process things, it does just feel like maybe in some ways you’re more efficient….. Maybe you can fit more appointments into the day, but I do think that’s at the expense of something else. And maybe slightly at the expense of your, I don’t know, not like your clinical competence, but just that kind of time of really processing things.*

*(Care Team Member)*

There was also the concern that time saving could become detrimental to participants themselves with an increased workload and changing schedules.

*I worry slightly about using more technology to make, to drive greater productivity out of our staff, because we always feel like we’re always running around like maniacs already trying to do too much. It’s always a bit of a concern when some people start thinking about cost and time saving.*

*(Consultant Geriatrician)*

## Discussion

Four interlinked themes were extracted from interviews with nine members of staff in a geriatric medicine department, which reflect the staff experiences of moving to remote care in a UK hospital. As the interviews took place from February to March 2021, interviewees had had 10–13 months’ experience of remote delivery. It is likely that the service itself evolved over this time as health services started to open up after the onset of the COVID-19 pandemic and remote consultations became more of a choice than a necessity. In presenting the findings, we have focussed on the aspects that present particular issues with this patient population, rather than challenges that are reported elsewhere (e.g., challenges with technology and equipment, considerations of which appointments are best suited to remote consultations). That is not to say that there are not issues with rapport, setting and the patient-professional relationship when conducting remote consultations with other patient groups, but rather that these present differently in geriatric medicine.

Theme 1 highlighted the challenges of remote consultations. Participants highlighted the difficulty of establishing rapport with patients remotely, particularly with phone rather than video calls. Many had been surprised that rapport *had* been possible to achieve, especially with patients who they had already met in person and had developed new techniques to promote a positive relationship. Nonetheless, there was a feeling that the rapport was not as strong or meaningful when only developed through remote meetings. Participants highlighted that remote consultations resulted in less informal interactions with patients and family members. These incidental interactions, which are not part of the traditional examination, are often important for providing an insight into aspects of the patient life that may not be obvious from asking the traditional assessment questions. In addition, participants highlighted how difficult it is to comfort patients remotely, therefore there was a strong feeling that the communication of bad news, a worsening condition, or an initial diagnosis should be done face-to-face.

Theme 2 highlighted participants’ perceived advantages of delivering care at a different location from the patient. Advantages included time saving, the ease at which patients’ families could be involved and a more comfortable experience for patients attending from their own homes. However, when staying at home, patients sometimes seemed less prepared or satisfied with what could be more informal appointments.

Theme 3 highlighted the disruption of involvement of family members during remote consultations. It was generally agreed that family members attending consultations are important for patient support and to help with communication and understanding. In some cases, remote consultations make it easier for family members, who live far away, to attend consultations, via a joint video or phone call. And this could be a positive step forward for care. However, joint calls meant a lack of opportunity for incidental or private conversations with family members and the care team.

Theme 4 outlined the impact on care staff, in particular the perceived changes in the patient-professional relationship, professional identity and confidence in doing the job well that had resulted from a shift towards remote consultations. Our participants reported a range of levels of confidence in delivering remote care which did not always relate to years of experience; for example, one consultant was very confident in making diagnoses remotely, whereas others admitted to requesting more tests to allay their concerns over the risk of missing something. For some, there was a feeling that remote consultations are just not appropriate to this patient group, whether this was due to frailty or age-related sensory impairments, the type of condition (especially cognitive deficits), or less familiarity with technology. Those who were against remote consultations for their patients considered remote care to be of a poorer, or less human and empathetic quality. The advantage of time saving associated with remote consultations was also seen as a disadvantage for the staff. In stable, non-complex, cases remote consultations do save time leading to seeing more patients, yet, reducing time for staff reflection, and increasing perceptions of care being more of a ‘production line’.

While it was sometimes hard to separate organisational changes (e.g., speed of discharge to community teams) from changes driven through the necessity to provide more remote care, it was clear that having a sustained and long-term relationship with patients was strongly valued by staff across different job roles, and that the quality of this relationship was perceived to be threatened as a result of service changes.

Within this relatively small sample, it was difficult to establish patterns in participant responses, to explore whether, for example, attitudes towards delivering remote care may be gendered, as well as relating to individual confidence in diagnosis and comfort in using technology. As one of our interviewees also commented, the type of health care professional who specialises in geriatric medicine may partly dictate their views; there was a feeling that the field may attract people who value building long-term relationships, which may not be so necessary in other medical fields, and thus those who may be less likely to embrace remote consultations regardless of patient group. The influence of these characteristics and factors would be interesting to explore. Previous literature reported that clinicians felt they missed out on physical examinations [20–22] and the communication with patients was not as good [14] in remote geriatric care. Perhaps this is more apparent in geriatric medicine because an important aspect of the traditional in person model is a comprehensive geriatric assessment, including both cognitive and physical examination [23], which can help strengthen the relationship between the clinician and patient [24, 25].

Many of the staff we interviewed considered geriatric patients to present a special need for face-to-face appointments due to their often complex health status incorporating both physical and cognitive co-morbidities. However, remote consultations did appear to be acceptable in some scenarios, and most care staff could see there was a role for them in parts of service delivery (and particularly for multi-disciplinary team meetings between staff). Remote consultations appeared more acceptable for triaging patients, to follow up or discharge a patient where a relationship has already been established and the patient is on a pre-arranged care pathway, and for those who are confident with technology and for whom meeting via video call may enable relatives who live further away to join in with a consultation. Initial visits requiring diagnosis, consultations with patients whose condition is not stable or who are not confident with technology, and appointments where a clinician or a member of the care team needs to break bad news, would not be acceptable.

## Limitations

This study is limited by providing the perspectives of a relatively small sample of nine staff from a single general hospital. While the single site provided the advantage that staff were reflecting on their experience of a similar setting, in terms of facilities, resources and patient group, including other sites may have flagged additional considerations and allowed reflection on potential influences of different environments or cultures. Nonetheless, we were able to elicit different perspectives of the same service both from within clinical roles, and between them. It was not always possible to separate service changes (e.g., to care pathways) from changes necessary to facilitate remote care; in some cases, tasks had been switched between nurses and consultants to reflect who would be meeting the patient in person, thus the format was driving the service rather than previous styles of good practice. In others, changes may have been independent of the COVID-19 response but conflated by staff as they took place during the same period.

## Conclusion

Moving towards a greater proportion of consultations delivered remotely was considered particularly problematic by staff working in geriatric medicine. While initially staff suggested this was as a result of patient and/or disease characteristics, it was clear that participant views of their role and expected depth and longevity of their relationship with patients also played a part. Some aspects of delivering remote care may threaten health care professionals’ perceptions of their ability to provide what they believe to be optimal, empathetic and patient-focused care in later life. This may mean that different models of implementing remote consultations, that allow for more of the relationship-based aspects of the role to be retained, are needed than in other medical disciplines, in order to be acceptable and rewarding for health care professionals working in the field.

## Supplementary Information

**Additional file 1.**

## Data Availability

The datasets used and/or analysed during the current study available from the corresponding author on reasonable request.
