# Supplementary material for: “A disembodied voice over the telephone”: a qualitative study of healthcare practitioners’ experiences in geriatric medicine
Source: BMC Geriatr. 2023 May 5;23:270. doi: 10.1186/s12877-023-03909-y (PMC10159677; doi:10.1186/s12877-023-03909-y)
Supplement: Supplementary file 1 — Additional file 1. [file 12877_2023_3909_MOESM1_ESM.docx]

**Appendix one.**

Remote consultations - Older Person Unit

Interview schedule - clinician

Thank you for agreeing to take part in this project. As you’ll have read in the information document, we are interested in your experience and views of remote Older Person Unit consultations. So, in this call, I’ll ask you some questions about these consultations – there are no right or wrong answers, we’re just interested in your opinions. I’ll record what is said in this call so that it can be typed up later, but any information that could identify you, such as names of people or places, will be removed. If at any time during the call you would like to stop, just let me know, and you do not have to answer any questions that you do not feel comfortable with.

[For Teams calls, may want to add something like: Just to let you know, I might be looking away from the camera at times – I will be paying attention! It’s just that I need to look at the screen to see the questions and the camera is in a different place.]

Before we begin, do you have any questions?

[Start recorder]

**To start with, can you tell me how you used to conduct typical face-to-face outpatient consultations?**

What preparation would you do before the consultation?

How would you start the consultation?

What information would you try to gather from the patient/family member/carer?

How would you try to end the consultation?

Are there any visual clues you look for in a face-to-face consultation, e.g., in the way a patient walks into the room or sits down?

**Can you tell me how you would conduct a typical remote consultation?**

What preparation would you do before the consultation?

How would you start the consultation?

What information would you try to gather from the patient?

How would you try to end the consultation?

What do you feel are the main differences between remote and typical face-to-face consultations from your perspective?

Have you noticed any differences in how patients respond in remote versus face-to-face consultations?

**What factors did you take into account when planning how you would run remote consultations?**

What formats did you consider?

Have you made changes since the outset?

Were you planning any move towards remote consultations before COVID, and if so what and why?

**What, if anything, have you found difficult in conducting remote consultations?**

Rapport building? Expressing empathy? Breaking bad news? Conducting cognitive assessments?

Understanding patients’ descriptions/building a clear picture of the problem without being able to see them? Assessing whether patients have understood advice?

Difficulty controlling the patient’s environment (e.g. interruptions from family members, noise etc.)?

Certain types of conditions or patient groups unsuitable for remote consultations?

Technology difficulties – poor lines, fuzzy pictures etc., or difficulties supporting the patient to use the technology

**Has anything helped you to overcome these difficulties or to improve your remote consultations?**

For example, has asking certain questions helped you to gain a better understanding of the patients’ conditions?

Has there been information you have sent out in advance which has helped to prepare the patient for the consultation?

Technology support improved connections?

**How have you found the use of PROMs to assess patients’ suitability for telephone consultations?**

Do they provide sufficient information?

Any concerns?

**Are there any advantages to remote consultations compared with consultations run in person?**

Time saving? Patients more relaxed/comfortable in their own homes?

**Would you have any concerns about the OPU continuing with, and potentially increasing the amount of, remote consultations in the long-term?**

For patient care, confidentiality or safety? (N.B. If the clinician is using cognitive assessments, they may have concerns about the validity of these assessments. Also, the validity of making diagnoses without seeing the patient.)

For clinical workload?

Would you be happy for more consultations to be conducted remotely in future (beyond the COVID pandemic)? Why/why not? Any provisos?

**What do you think are the markers of a successful or good quality remote consultation?**

And what would characterise a poor quality remote consultation?

**If we wanted to measure the impact of conducting consultations remotely, what do you think we should measure?**

**Is there any support or training, for clinicians or patients, that you think would improve the quality of remote consultations?**

Are the protocols you have for face-to-face appointments (e.g., breaking bad news etc) fit for purpose with remote consultations?

Help for patients in setting up video-call equipment, access to more advanced technology, guidance for clinicians on effective consultations?

That’s all my questions, is there anything else you would like to add?

Thank them for their time and stop recorder.

Remote consultations – Older Person Unit

Interview schedule – non-clinical staff

Thank you for agreeing to take part in this study. As you’ll have read in the information document, we are interested in your experience and views of remote Older Person Unit (OPU) consultations. So, in this call, I’ll ask you some questions about these consultations – there are no right or wrong answers, we’re just interested in your opinions. I’ll record what is said in this call so that it can be typed up later, but any information that could identify you, such as names of people or places, will be removed. If at any time during the call you would like to stop, just let me know, and you do not have to answer any questions that you do not feel comfortable with.

Before we begin, do you have any questions?

[Start recorder]

**To start with, can you explain to me your role in the remote consultations in the OPU?**

And how does this differ from your role in typical face-to-face consultations?

**What, if anything, have you found difficult with regards to the remote consultations?**

Technology difficulties?

Understanding or working within data protection/security regulations?

Patient/clinician opposition?

**Has anything helped you to overcome these difficulties?**

Technology support improved connections?

Conversations with/training of staff/patients?

**How have you found the use of PROMs to assess patients’ suitability for telephone consultations?**

Any concerns?

**Are there any advantages to remote consultations compared with consultations run in person?**

Time saving? Patients preference?

**Would you have any concerns about the OPU continuing with, and potentially increasing the amount of, remote consultations in the long-term?**

For patient care, confidentiality or safety?

For clinical/administrative workload?

Would you be happy for more consultations to be conducted remotely in future (beyond the COVID pandemic)? Why/why not? Any provisos?

**If we wanted to measure the impact of conducting consultations remotely, what do you think we should measure?**

**Is there any support or training, for clinicians, non-clinical staff or patients, that you think would improve the quality of remote consultations?**

For example, help for patients in setting up video-call equipment, access to more advanced technology, guidance for clinicians on effective consultations?

That’s all my questions, is there anything else you would like to add?

Thank them for their time and stop recorder.
